# Unintended Pregnancies among Young Women Living in Urban Slums: Evidence from a Prospective Study in Nairobi City, Kenya

**DOI:** 10.1371/journal.pone.0101034

**Published:** 2014-07-31

**Authors:** Donatien Beguy, Joyce Mumah, Lindsey Gottschalk

**Affiliations:** 1 African Population and Health Research Center, Urbanization and Wellbeing and Population Dynamics and Reproductive Health Programs, Nairobi, Kenya; 2 Johns Hopkins School of Public Health, Baltimore, Maryland, United States of America; Columbia University, United States of America

## Abstract

**Background:**

Despite the significant proportion of young people residing in slum communities, little attention has been paid to the sexual and reproductive health (SRH) challenges they face during their transition to adulthood within this harsh environment. Little is known about the extent to which living in extreme environments, like slums, impact SRH outcomes, especially during this key developmental period. This paper aims to fill this research gap by examining the levels of and factors associated with unintended pregnancies among young women aged 15–22 in two informal settlements in Nairobi, Kenya.

**Methods:**

We use data from two waves of a 3-year prospective survey that collected information from adolescents living in the two slums in 2007–2010. In total, 849 young women aged 15–22 were considered for analysis. We employed Cox and logistic regression models to investigate factors associated with timing of pregnancy experience and unintended pregnancy among adolescents who were sexually active by Wave 1 or Wave 2.

**Findings:**

About two thirds of sexually experienced young women (69%) have ever been pregnant by Wave 2. For 41% of adolescents, the pregnancies were unintended, with 26% being mistimed and 15% unwanted. Multivariate analysis shows a significant association between a set of factors including age at first sex, schooling status, living arrangements and timing of pregnancy experience. In addition, marital status, schooling status, age at first sex and living arrangements are the only factors that are significantly associated with unintended pregnancy among the young women.

**Conclusions:**

Overall, this study underscores the importance of looking at reproductive outcomes of early sexual initiation, the serious health risks early fertility entail, especially among out-of school girls, and sexual activity in general among young women living in slum settlements. This provides greater impetus for addressing reproductive behaviors among young women living in resource-poor settings such as slums.

## Background

Approximately one-third of the African population is between the ages of ten and twenty-four, the highest proportion of youth anywhere in the world [1], [2]. This means that one-third of the population in this region is in the process of transitioning to adulthood, a transition that includes, among other monumental changes, the beginning of a person’s sexual and reproductive life [3]. It is during this transitional period that many young women in sub-Saharan Africa (SSA) first enter into motherhood. SSA has the highest level of adolescent fertility in the world, which significantly contributes to the region’s lifetime average of 5.1 births per woman [4], [5].

Though childbearing is a natural part of the transition to adulthood, evidence shows that a significant portion of adolescent fertility is unintended–either unwanted or mistimed–across countries in SSA [6]. Unintended adolescent pregnancy poses a clear public health challenge, as both unintended pregnancy and pregnancy at a young age are associated with adverse health outcomes for mother and newborn. It is estimated that one-third of all unintended pregnancies in Africa end in induced abortion [7]. This is disconcerting given that the majority of abortions in the region are performed under unsafe conditions due to restrictive abortion laws, lack of knowledge, and inaccessibility of services [6]–[8]. Young women 15–24 account for over half of all unsafe abortions as well as over half of all maternal deaths from unsafe abortions in the region [9], [10]. About a quarter of the growing number of deaths among 10–24 years old women in SSA is due to maternal mortality [11]. Among women who carry their unintended pregnancies to term, adolescents face an increased risk for obstetric complications such as obstructed labor, anemia, and preeclampsia, which can result in long-term morbidities and even death [12]–[14]. Evidence also suggests that women with unintended pregnancies are less likely to seek appropriate prenatal care, placing them at higher risk for complications before and after delivery [15]–[17]. This lack of appropriate prenatal care has implications for the children born to adolescent parents, as they tend to have higher incidences of low birth weight, prematurity, stillbirth, and neonatal mortality [18]–[21]. In addition to acute health risks, adolescent mothers may also face social consequences of unintended pregnancy, including poor educational and employment prospects, marital instability, single parenthood, social ostracism, and an increased risk of getting into or remaining in poverty [4], [22].

The population of sub-Saharan Africa is growing faster than anywhere else in the world, and the strains of population growth are increasingly felt in the region’s urban areas. As sub-Saharan Africa undergoes rapid urbanization, the population of adolescents transitioning to adulthood in informal settlements–or “slums”–is quickly rising [23]–[27]. Slum communities are characterized by a lack of basic infrastructure, poor schooling facilities, high risk of sexual and gender-based violence, high levels of substance abuse and poor livelihood opportunities, all of which negatively impact the health and wellbeing of residents [28]–[30]. Even with overall reductions in the proportion of the SSA population living in poverty, the slums in SSA continue to grow at an alarming rate. Between 1990 and 2010, the proportion of urban residents in SSA living in slum settlements declined from 70 to 62%, yet the population of slum dwellers doubled from 103 to 200 million [27].

Despite the significant proportion of adolescents residing in slum communities, little attention has been paid to the sexual and reproductive health (SRH) challenges they face during their transition to adulthood within this harsh environment. While it is well established that there are social determinants of SRH–which may include education, poverty, social norms, and health systems–little is known about the extent to which living in extreme environments, like slums, impacts SRH, especially during this key developmental period [31]. Studies of adolescents living in informal settlements in Bangladesh, Brazil and Kenya provide some of the few insights into the reproductive health challenges facing this population and suggest that adolescent girls in these communities are especially at risk for unplanned pregnancies given poor education, insecurity within the community, and low-levels of autonomy stemming from cultural norms and the desperation of poverty [32]–[35]. Evidence from slum communities in Nairobi indicate that adolescents living in the slums engage in riskier sexual behaviors than their peers in non-slum parts of cities, including early sexual debut, transactional sex, and multiple sexual partnerships [28], [35], [36]. Additionally, adolescents’ knowledge about contraception is inadequate, and access to contraceptive methods is limited in slum communities, impinging an adolescent girl’s ability to control her reproductive life [37]. This is especially troubling, since adolescents are already at a disadvantage when it comes to accessing contraceptive services in SSA, independent of poverty. In Kenya, for example, 45% of all sexually active unmarried adolescents have an unmet need for contraception [38]. Because the urban poorest population tends to have the greatest unmet need, these estimates are likely even greater among adolescents in the slums [37].

There are adolescents, however, that transition to adulthood in the slums without experiencing a major adverse reproductive health event. Studies from Brazil and Kenya suggest that there are protective factors, such as having a father present in the home, that are associated with delayed sexual debut and fewer incidences of unintended pregnancy among adolescents in slum communities [34], [39]. Further research may be able to elucidate additional factors that help adolescents in these communities lead healthy and fulfilling sexual and reproductive lives.

This paper seeks to expand on this limited body of research by examining the factors associated with unintended pregnancies among young women aged 15–22 living in two informal settlements in Nairobi city, Korogocho and Viwandani. The use of prospective data allows us to investigate the association between pregnancy experience and status between two waves of data collection and various relevant factors captured during the first wave. Unlike other similar studies, we therefore control for the temporal ordering of the explanatory factors and the outcome variables. The findings may help in understanding the circumstances and factors surrounding unintended pregnancies among young people in order to inform policy development, design and implement appropriate interventions, and identify areas for further research. Understanding and identifying ways to address the poor reproductive health outcomes among adolescents and young adults is central to the attainment of the Millennium Development Goals (MDGs) and other development indicators in Kenya and other sub-Saharan African countries. As urbanization continues unabated in the region, the wellbeing of the urban poor will increasingly drive national development indicators. Preventing unintended pregnancies among young women would greatly reduce maternal and infant mortality and disability adjusted life years (DALYs) among this vulnerable age group, increase educational and livelihood opportunities for girls, and ease the strain of population growth on communities and nations. Beyond reducing their adverse health consequences, averting unintended pregnancies among adolescents and young female adults living in urban slums could also contribute to slowing rates of urban growth which is substantially brought about by natural increase in SSA [40]. Ezeh et al. [37] show that in Kenya and other countries in SSA, mistimed and unwanted fertility significantly contribute to the high fertility among the urban poor.

### Study context

Korogocho and Viwandani slums are located about 7 kilometers from each other, and are located about 12 and 7 kilometers from the central business district, respectively. Though both slums are in some of the most congested slum areas in Nairobi city, there are remarkable differences in terms of demographic, economic and health dynamics between the two communities. In terms of population, Korogocho is home to a stable community which has settled there for many years. On the contrary, Viwandani, which is located in the industrial area, attracts a highly mobile population in search of employment in the industries. The duration of residence among migrants is higher in Korogocho, corroborating the relative stability of the population in Korogocho compared to Viwandani [41]. The majority of individuals are migrants although the proportion is much higher in Viwandani compared to Korogocho (95% vs. 75%). The majority of migrants moved into the slums when they are young adults. Between 13% and 24% migrated into the two slums while aged 15–19 years. Korogocho exhibits a higher dependency ratio, with Viwandani attracting more adult males (15–64) than Korogocho. Previous findings show that 18% of young women aged 12–22 entered into first union by age 18. By age 22, 34% of these young women were already married or living with a partner. However, for 77% of them, sexual debut occurred outside of marriage or other union [3]. Furthermore, another study indicates that 16% of females aged 10–19 living in Kibera slum in Nairobi reported that they had ever been married [42].

## Data and Methods

### Data

We draw from data collected between 2007 and 2010 under a 3-year prospective cohort study – the Transitions to Adulthood Project (TTA), which was part of the larger Urbanization, Poverty, and Health Dynamics (UPHD) research program. The UPHD was nested into the Nairobi Urban Health and Demographic Surveillance System (NUHDSS), a longitudinal platform set in 2002 by the African Population and Health Research Center to collect and monitor health and demographic data from residents living in Korogocho and Viwandani slums. The TTA study follows a cohort of randomly selected NUHDSS residents aged 12–22 years (at baseline) to identify protective and risk factors in their lives, and to examine how these factors influence various markers of the transition to adulthood. The Kenya Medical Research Institute’s ethical review board provided ethical approval for the TTA study.

### Study participants, procedures

The baseline survey was conducted between October 2007 and June 2008. The second and third rounds were conducted between March 2009 and August 2009, April 2010 and August 2010, respectively. Duration between rounds of surveys ranged between 9.6 and 21.6 months. The surveys collected information on, among other details, socio-demographic characteristics; parent-child relationships, sibling and other influence, domestic turbulence and sexual abuse, self-esteem, peer influence, and delinquent behavior (questions on drug use and depression added in Wave 2), concerns, aspirations, and expectations or perceived life chances, circumcision, marriage and dating, sexual behavior, contraceptive use, childbearing, and childbearing aspirations, HIV/AIDS-related knowledge and HIV testing, attitudes towards sex and contraceptive use (Attitudes to condom use added in Wave 2) and civic participation. In addition, we administered a life history calendar to capture transitions in schooling, independent housing, marital status, sexual intercourse, pregnancy, and income generation. For each survey, the questionnaires were pilot-tested among adolescents in areas within the two slums that were not covered by the routine NUHDSS data collection. The questionnaire was mainly administered in Kiswahili, the lingua franca in the two slum areas, after original and translated versions were reviewed by language professionals to ensure comparability.

In total, 4,058 youth (50% males) aged 12–22 were interviewed during the baseline survey. In Wave 2, 2,674 young people were interviewed. Overall, refusals were low (<5%) among youth whom fieldworkers were able to reach and the relatively low re-interview rate is mainly attributable to difficulties in locating some of the youth given the high mobility of residents in the area [43]. In this study, we limit the sample to 849 young women aged 15–22 who were observed at both Waves 1 and 2 of data collection, and who had non-missing data on the variables used for our analyses.

### Measures

The first outcome variable is pregnancy experience as measured by the response to the question “Since our last visit, have you been pregnant?” that was asked during Wave 2. A binary variable indicating whether a pregnancy occurred or not (Yes vs. No) is derived from this question to capture young people’s pregnancy experience. The second outcome variable is the pregnancy intention as measured by young women’s answers to direct questions on last pregnancies during the three survey rounds. Young women who were not pregnant at the time of the survey were asked: “At the time you became pregnant the last time, did you want to become pregnant then, did you want to wait until later, or did you not want to have any (more) children at all?”. For those who were pregnant at the time they were interviewed, they responded to the question: “For your current pregnancy, did you want to become pregnant, did you want to wait until later, or did you not want to have any (more) children at all?” The answers were “then”, “later” and “not at all”. The variable pregnancy intention has 3 categories: intended, mistimed and unwanted. Intended pregnancy is a pregnancy that was planned by the woman. Mistimed pregnancy is a pregnancy for which a woman reported wanting to waiting until later before getting pregnant, while unwanted pregnancy refers to a pregnancy for which a woman indicated that she did not want any more children at all. Mistimed and unwanted pregnancies constitute unintended pregnancies. To note, for pregnancies that are already terminated, the planning status is reported retrospectively at the time of conception and not at the time of the survey, suggesting that it might be affected by recall bias or “ex post-facto rationalization” by respondents.

We include in our regression analysis the following socio-demographic characteristics measured at Wave 1: slum area of residence (Korogocho vs. Viwandani), age group (15–17 vs. 18–22), educational level (primary or lower vs. secondary or higher), ethnic group (Kikuyu, Luyha, Luo, Kamba, Somali, Other ethnic groups), religion (No religion, Catholic, Other Christian and Muslim), schooling status (in school vs. out of school). Other characteristics associated with first sex were also considered: age at first sex (less than 15, 15–17 and 18–23); relationship with first sexual partner which has three categories (husband/partner, boyfriend and other); contraceptive use at first sex (Yes vs. No). For socio-economic status, adolescents are grouped into three categories based on a wealth index computed using principal component analysis: poor, middle, rich. We also included a variable capturing parental presence in the young people’s life: living with mother only, father only, both parents, neither parent.

### Analytical approach

All analyses were conducted using Stata 12.1 [44]. We provide descriptive findings on sexual experience, pregnancy experience and pregnancy intention using univariate statistics. We used survival analysis through the Cox regression model to investigate the influence of various factors on the timing of pregnancy experience between the two waves [45]–[47]. Women were considered to be at risk from first wave until they become pregnant or censored at the time of the survey for those who were still not pregnant. Hazard ratios are obtained from Cox model. A hazard ratio greater than one indicates higher risk of pregnancy experience. Conversely, the risk is lower when the hazard ratio is less than one. On the other hand, we employed multivariable logistic regression to investigate factors associated with unintended pregnancy among adolescents who were sexually active at Wave 1 or by Wave 2. To increase the size of the sample for analysis, we included in the multivariate analysis young women who have not initiated sex by Wave 1 but have become sexually active by Wave 2. For these young women, some of the explanatory factors such as use of contraception at first sex, relationship with first partner and age at first sex were captured retrospectively at Wave 2. To control for the temporal ordering of the explanatory factors and outcome variables, we use information collected at Wave 1 (baseline) or between Wave 1 and Wave 2 to explain pregnancy experience/intention in Wave 2. To account for potential lack of independence for adolescents living in the same household, we adjusted estimates for the standard errors using clustered sandwich estimator (Huber/White/sandwich estimate of variance) [48], [49] implemented in Stata 12.

Of the 1,378 adolescent girls aged 15–22 interviewed at Wave 1, 849 (62%) were re-interviewed at Wave 2. Findings from a model predicting attrition between Wave 1 and Wave 2 among this sub-sample indicate that adolescents aged 20–22, those from Luhya and Luo ethnic groups were more likely to be lost to follow-up. On the contrary, those still in school, and those living with at least one parent father were less likely to drop out between the two surveys (results not shown). We therefore computed weights to adjust for attrition between Wave 1 and 2.

### Ethics statement

The Kenya Medical Research Institute’s ethical review board provided ethical approval for the TTA study. We obtained written or verbal consent from all study participants. For respondents who are willing to participate in the survey but could not or were not willing to sign the consent form, verbal consent was obtained and the field interviewer had to note on the consent form that the participant was willing to participate but unable to sign the consent form. Parental consent was also obtained for those aged 12–17 years. The consent form and process were approved by the KEMRI ethical review board.

## Results

### Descriptive findings

#### Sample description

Table 1 presents some key socio-demographic characteristics of the study participants at the time of the Wave 1 survey. In total, 849 girls aged 15–22 observed at Waves 1 and 2 are considered for analysis in this paper. About 78% are aged 15–19, a bit more than half (53%) are from Viwandani, 72% are Catholic or Other Christian; and 78% have never been married. In addition, 49% were still in school at the time of Wave 1 survey; 47% have reached at a secondary level of education.

**Table 1:** Percentage of female adolescents (15–22) who reported having initiated sex, by socio-demographic characteristics (as of Wave 1).

Socio-demographics	All			Unmarried		
	N	% total	% Everhad sex	N	% total	% Everhad sex
Total	849	100.0	37.3	665	100.0	25.3
*Age group*						
15–19	658	77.5	26.4	566	85.1	20.2
20–22	191	22.5	74.7	99	14.9	54.3
*Slum area* *of residence*					ss	
Korogocho	402	47.3	39.0	336	50.5	32.3
Viwandani	447	52.7	35.7	329	49.5	18.1
*Socio-economic* *status*						
Poor	290	34.2	39.1	226	34.0	26.8
Middle	203	23.9	33	165	24.8	23.2
Rich	356	41.9	38.2	274	41.2	25.3
*Religion*						
None	95	11.1	52.9	66	9.9	38.5
Catholic	224	26.3	44.7	169	25.4	32.3
Other Christian	384	45.3	36.7	308	46.3	26.2
Muslim	147	17.3	17.4	121	18.2	6.1
*Education level*						
Primary	453	53.4	43.7	333	50.1	28.7
Secondary or higher	396	46.6	29.9	332	49.9	21.8
*Schooling status*						
No	433	51.0	62.7	276	41.5	45.5
Yes	416	49.0	10.9	389	58.5	10.9
*Marital status*						
Never married	708	83.4	25.3		-	-
Currently married	141	16.6	97.5		-	-
*Ethnic group*						
Kikuyu	335	39.5	38.8	271	40.8	29.1
Luhya	81	9.5	50.7	64	9.6	41.5
Luo	100	11.7	50.6	77	11.6	40.2
Kamba	139	16.4	39.9	97	14.6	22.2
Other	194	22.9	20.4	155	23.3	6.3
*Parental presence*						
Mother only	23	2.7	37.3	21	3.2	34.3
Father only	235	27.7	31.4	213	32.0	29.8
Both parents	419	49.3	21.3	383	57.6	19.3
Neither	172	20.3	84.2	49	7.4	48.6

#### Sexual initiation

Findings indicate that about 37% of young women aged 15–22 have already initiated sex in their lives, with the proportion being significantly higher among those aged 20–22 (Table 1). Young women living in Viwandani are marginally less likely to report having initiated sex; 36% compared to 39% of their Korogocho counterparts reported sexual debut. Young women who are still in school are less likely to have initiated sex; the proportion of those who have already experienced sex is lower among those with at least a secondary level of education (30% vs. 44%). When considering religion, Muslims exhibit the lowest proportion of those who have initiated sex. With respect to ethnic group, the highest percentage of sexually experienced is observed among young women from Luo and Luyha ethnic groups. As expected, the proportion of those who reported sexual debut is much higher among currently married young women (98% vs. 25%). Young women who live with neither parent are much more likely to have initiated sex. As indicated in Table 1, similar trends of bivariate associations are observed among unmarried female youth. Results on the sexual experience by parental presence among the married women (not shown) indicated that the majority of them do not live with their parents (85%), and that 99% of those young women have initiated sex.

#### Pregnancy experience and pregnancy intention

Table 2 shows data on young women’s pregnancy experience and their pregnancy intention at Wave 2 of young women who have already initiated sex by Wave 1 or between Wave 1 and 2, by socio-demographics. About two-thirds of sexually experienced female youth (69%) have ever been pregnant by Wave 2, with the percentage being higher among those aged 20–22. The proportion of those who have already been pregnant is higher among those with primary education. Ever-married young women are significantly more likely to report having experienced a pregnancy (93%) compared with those who have never been married (55%). Those who have had their first sexual experience with their husband/partner, or never used contraception at first sex are proportionately more likely to have ever been pregnant. The percentage of ever pregnant young women is higher among those who live with neither parent, and those who have initiated sex before age 15.

**Table 2:** Pregnancy experience and pregnancy intention for young women aged 15–22 who have ever been pregnant.

Socio-demographics	Ever pregnantat Wave 2		Pregnancy intentionat Wave 2			
	N	%	N	% Intended	% Mistimed	% Unwanted
Total	471	69.2	316	59.2	25.9	14.9
*Age group*						
15–19	255	60.4	148	48.6	34.5	16.9
20–22	216	79.6	168	68.5	18.5	13.1
*Slum area of residence*						
Korogocho	222	63.5	139	42.4	36.7	20.9
Viwandani	249	74.3	177	72.3	17.5	10.2
*Socio-economic status*						
Poor	180	65.6	115	60.9	27.8	11.3
Middle	121	70.2	81	55.6	29.6	14.8
Rich	170	72.4	120	60	21.7	18.3
*Religion*						
None	71	71.8	50	60	28	12
Catholic	141	68.8	92	60.9	23.9	15.2
Other Christian	230	66.1	150	54.7	29.3	16
Muslim	29	89.7	24	79.2	8.3	12.5
*Education level*						
Primary	277	78.7	212	57.1	26.4	16.5
Secondary or higher	194	55.7	104	63.5	25	11.5
*Marital status*						
Never married	298	55.4	160	39.4	37.5	23.1
Currently married	173	93.1	156	79.5	14.1	6.4
*Ethnic group*						
Kikuyu	177	65.5	113	52.2	31	16.8
Luhya	66	72.7	46	52.2	26.1	21.7
Luo	82	61.0	49	44.9	36.7	18.4
Kamba	92	71.7	63	69.8	20.6	9.5
Other	54	85.2	45	84.4	8.9	6.7
*Relationship with first partner*						
Husband/Partner	113	86.7	95	81.1	10.5	8.4
Boyfriend/Other	358	63.7	221	49.8	32.6	17.6
*Age at first sex*						
Less than 15	73	74.0	51	54.9	29.4	15.7
15–17	244	71.7	172	52.9	29.7	17.4
18–23	154	63.0	93	73.1	17.2	9.7
*Contraceptive use at first sex*						
No	255	78.4	192	60.9	24	15.1
Yes	216	58.3	124	56.5	29	14.5
*Parental presence*						
Mother only	10	40	3	100	0	0
Father only	103	56.3	55	30.9	43.6	25.5
Both parents	147	53.1	75	34.7	40	25.3
Neither	211	88.2	183	77	15.3	7.7

In addition, findings show that overall, 41% of pregnancies were unintended, with 26% being mistimed and 15% unwanted. The percentage of young women with unintended pregnancy is estimated at 51% and 31% for 15–19 and 20–22 years old, respectively. Adolescents who have never been married exhibit higher levels of unintended pregnancy (61% vs. 20% for those who are currently or formerly married). The proportion of unintended pregnancy is higher among those who had their first sexual encounter with their boyfriend as compared with those who first experienced sex with their husband/partner (50% vs. 19%). In addition, the percentage of those who have had an unintended pregnancy is higher among those who initiated sex before age 18 and those who live with at least one parent.

### Multivariate analysis

Results from multivariate analysis for timing of pregnancy experience (Cox model) and pregnancy intention (logistic regression) are presented in Table 3. Estimations indicate that being in school is negatively associated with the timing of pregnancy experience as young women who are still in school are significantly less likely to have had a pregnancy. Young women from Luhya ethnic group get pregnant earlier as they are as twice as likely to become pregnant compared with their Kikuyu counterparts. Early initiation of sex increases the chances of pregnancy as those who initiated sex before age 18 have had a pregnancy episode earlier than those who experienced first sex from age 18. Young women who live with their mother only or both parents experience a pregnancy later than those who live with neither parent. In addition, marital status, schooling status, age at first sex and living arrangements are the only factors that are significantly associated with unintended pregnancy among these young women. In fact, young women who have initiated sex before age 18 are more likely to experience an unintended pregnancy but the coefficient is statistically significant only for those whose sexual debut occurred between ages 15 and 17. Also, being in school reduces the chances of experiencing an unintended pregnancy (at 10% level). Young women who were married are much less likely to have had an unintended pregnancy. Interestingly, adolescents living with their father only or both parents are much more likely to have had an unintended pregnancy than their counterparts who live with neither parent.

**Table 3:** Factors associated with timing of pregnancy experience (Cox regression) and unintended pregnancy among young women aged 15–22 (Logistic regression).

Socio-demographics	Hazard ratios (95% CI) (Cox regression model)	Odd ratios (95% CI) from(Logistic regression model)
	Becoming pregnant(Among those who initiatedsex by Wave 1 or Wave 2)	Had an Unintended pregnancy(Among those ever pregnant at Wave 2)
*Age group*		
*15–17*	Ref.	Ref.
18–19	0.72 (0.48–1.09)	0.59 (0.21–1.65)
20–22	1.00 (0.66–1.50)	0.60 (0.21–1.74)
*Slum area*		
Korogocho	Ref.	Ref.
Viwandani	0.82 (0.61–1.10)	0.61 (0.31–1.21)
*Wealth status*		
Poor	Ref.	Ref.
Middle	1.26 (0.90–1.77)	1.11 (0.54–2.26)
Rich	0.89 (0.66–1.20)	1.01 (0.50–2.01)
*Religion*		
None	Ref.	Ref.
Catholic	1.35 (0.90–2.01)	0.84 (0.37–1.94)
Other Christian	1.21 (0.85–1.72)	1.08 (0.50–2.33)
Muslim	1.14 (0.68–1.91)	1.32 (0.22–7.84)
*Education*		
Primary	Ref.	Ref.
Secondary or higher	0.95 (0.72–1.26)	0.94 (0.48–1.83)
*Currently in school*	0.41** (0.19–0.91)	0.32* (0.10–1.02)
*Currently married*	1.03 (0.69–1.53)	0.34** (0.15–0.79)
*Ethnic group (Kikuyu)*		
Kikuyu	Ref.	Ref.
Luhya	2.13*** (1.43–3.18)	1.06 (0.46–2.46)
Luo	0.91 (0.64–1.31)	1.74 (0.76–3.96)
Kamba	1.06 (0.70–1.61)	0.95 (0.40–2.27)
Other	1.22 (0.84–1.78)	0.28* (0.07–1.16)
*Relationship with first sex partner*		
Boyfriend/Other	Ref.	Ref.
Husband/Partner	1.09 (0.79–1.52)	0.82 (0.39–1.75)
*Age at first sex*		
Less than 15 years	1.54** (1.05–2.25)	1.11 (0.40–3.10)
15–17	1.30* (0.97–1.75)	2.17** (1.02–4.58)
*18–23*	Ref.	Ref.
*Used contraceptive at first sex*	1.01 (0.72–1.41)	1.33 (0.73–2.42)
*Parental presence*		
Neither	Ref.	Ref.
Mother only	0.37** (0.17–0.81)	-
Father only	1.12 (0.69–1.82)	3.13** (1.14–8.59)
Both parents	0.55** (0.35–0.87)	3.21** (1.28–8.02)
Wald Chi-square	47.93***	74.43***
Person-months or N	5419.2	313

Significant at p<0.01*** p<0.05** p<0.1*.

## Discussion

In this paper, we use data from baseline and second round survey of a three-year prospective study, to examine the pregnancy experience and intention of young women aged 15–22 in two informal settlements in Nairobi. About two-thirds of sexually experienced adolescents have ever been pregnant by Wave 2. For 41% of adolescents, the pregnancies were unintended, with 26% being mistimed and 15% unwanted. Findings indicate a significant association between a set of factors including age at first sex, schooling status, living arrangements and timing of pregnancy experience. In addition, marital status, schooling status, age at first sex and living arrangements are the only factors that are significantly associated with unintended pregnancy among the young women. These findings contribute to filling the gap in understanding and meeting the sexual and reproductive challenges and needs of young women living in resource-poor urban settings such as informal settlements in the country.

Overall, our findings show that early sexual initiation was prevalent as about 37% of female youth aged 15–22 had already initiated sexual intercourse, though the proportion of sexually experienced adolescents vary significantly with age, marital status and schooling status. Young women were less likely to initiate sex if they were currently in school, with at least a secondary education and never married. Most notably, the odds for young women currently in school reporting a pregnancy experience are substantially lower than for those who are out-of-school, suggesting that pregnancy may lead to school drop-out among young women. Earlier studies in Kenya have reported that 13,000 girls drop out of school annually due to early childbearing [50]. Although school environment seems to be protective against childbearing, it may also be true that both drop-out and pregnancy are influenced by a same set of factors. This findings call for more school-based studies to help disentangle the association. This result mirrors studies done in other African context were initiation of first sex (before age 18) was less likely during adolescence if the girl was educated or exposed to mass media [35], [51]. Similarly, other studies indicated the role of education in reducing fertility in Kenya and elsewhere in sub-Saharan Africa [4],[52]. This indicates that keeping girls in school and formal education in general might be an effective strategy to enable young girls delay sexual debut. Increased education might therefore act as a protective mechanism that not only prevents early sexual initiation, but more education improves the understanding by young women of the need to protect themselves from unintended pregnancy.

Moreover, about two-thirds of girls with previous sexual experience had experienced a pregnancy; 41% of them reported having had an unintended pregnancy. In addition, our results indicated that age at first sexual intercourse was associated with timing of pregnancy experience on one hand and pregnancy intention on the other. Young women whose sexual debut occurred before age 18 got their pregnancy earlier than those who initiated sex from age 18. In addition, young women who had initiated sexual intercourse between the ages of 15–17 had significantly higher odds of experiencing an unintended pregnancy, indicating that early sexual initiation was associated with adverse SRH outcomes. Early sexual debut is much likely to automatically increase young women’s exposure to the risk of pregnancy, especially in the slum settlements where use of contraception is limited. More than often, young people’s first sexual experience is unplanned, a pattern that predisposes them to irregular and ineffective contraceptive use. Extant literature indicates that premarital sexual activity among adolescents is increasing but less than half are using any modern contraception to avoid pregnancy [53], [54]. It is reported that barriers to accessing health care such as lack of decision-making power, access to and control over resources, and socio-cultural norms regarding adolescent sexual behavior and childbearing contribute to low use of contraception [55]. Moreover, the nature of services offered, who and the attitude of who is offering the services reportedly influences young people’s attitude toward contraceptive use, as well as their ability to express their needs and consequent health seeking behavior [56]. Provider bias and attitude toward adolescent therefore discourages young people from accessing essential contraceptive information and services. This finding highlights the fact that targeted programs are needed to reach adolescents and young adults with a range of SRH information and services at different stages - before they initiate sex or as sexually active unmarried adolescents. Moreover, reproductive health interventions including uptake of family planning which often do not reach adolescents, should be scaled up to include adolescent friendly services, with targeted responses required. Given that schooling was shown to be protective, bringing comprehensive sexuality education into schools and moving beyond the fear that sex education for young people encourages risky behaviors is important. In fact, previous studies show that providing sex education to young people in schools has the potential to be protective, especially in delaying early sexual initiation [57].

Findings also indicate that ethnicity had statistically significant effect on timing to pregnancy between Wave 1 and 2; young women from Luhya ethnic group are more likely to become pregnant than those from Kikuyu tribe. This finding is consistent with higher levels of fertility observed in Western Kenya where Luhyas come from. This may partly due to Kikuyu women’s higher access to education, higher involvement in economic activities that empower them to have a better control over their reproductive lives. Also, Kikuyus are known to be less pronatalist than their counterparts from Luo and Luhya ethnic groups [29], [58], [59].

Living with a mother only or both parents reduced the risks of becoming pregnant. Evidence from other SSA settings indicates that parental monitoring and control reduces the likelihood of involvement in problem behavior and is likely to lead to delayed onset of sexual experience [60], [61]. In particular, living with both parents is associated with lower risk of sexual experience, and therefore lower chances of getting pregnant. It also appears that having the mother in the household is important in preventing pregnancy experience, suggesting that the father’s absence does not deter the mother’s ability to control her daughter’s activities. However, on the other hand, living with a father only or both parents was significantly associated with higher chances of experiencing an unintended pregnancy. In a study on transition to first sex by adolescents in Kenya, Kabiru et al. [35] found that high level of parental monitoring was associated with greater odds of transitioning to first sex for females in the slums, which is positively associated with higher levels of unintended pregnancy. It is noted that the social context of slum environment especially cramped living conditions and shared living space, might expose children to early sexual activity [62]. Furthermore, perception by adolescents of parents being overly controlling has been shown to instead increase risky sexual behavior [63]. Our findings therefore highlight the fact that ways to adequately involve parents in sexual and reproductive health interventions targeted towards adolescents must be explored and tested, especially in resource-poor settings such as slums.

Current marital status is significantly associated with unintended pregnancy, with currently married adolescents being much less likely to have had an unintended pregnancy. However, adolescent childbearing is associated with increased risk of adverse health outcomes, regardless of whether a pregnancy is intended or not. Adolescent fertility still has significant implications not only for the health of the adolescent girl but also for larger demographic processes, especially high fertility rates in sub-Saharan Africa. It is reported that adolescent girls are more likely to die during pregnancy and delivery, because of their physical immaturity [64]. Moreover, younger mothers are also more likely to have babies with significant mortality and morbidity risk. For example, a study of 76 countries showed children born to mothers between the ages of 12–20 had a greater risk of dying before age five, being stunted, underweight or suffering from anemia [64]. Overall, this study underscores the importance of looking at reproductive outcomes of early sexual initiation, the serious health risks early fertility entail, especially among out-of school girls, and sexual activity in general. This provides greater impetus for addressing adolescent and young females’ reproductive behaviors, especially in resource-poor settings such as slums. In particular, it is paramount to identify and dismantle barriers that prevent adolescents and young women living in slum areas, from accessing existing family planning services.

While the prospective nature of the data used helps partially overcome the causality issue that is often encountered when using cross-sectional data, findings in this paper should be interpreted in light of some limitations. For instance, given the relatively short duration between the two waves of data collection, not many episodes of pregnancy were observed among adolescents. As a result, the analysis of pregnancy experience and status is based on a relatively small number of cases. In addition, our analysis could be affected by unobserved potential confounding variables that may influence both the observed explanatory variables and pregnancy experience/status. Finally, retrospective self-reporting of the status of pregnancies might be biased as mother’s feelings about pregnancy might change over time.

Despite these inherent limitations, the findings of this study have far-reaching implications for reproductive health of poor adolescents and young women in Kenya. Two clear policy and programmatic implications arise from our findings. First, it is important to design appropriate and realistic programs that are focused on reducing the number of unintended pregnancies; thereby helping reduce the ensuing induced abortions among adolescents and young women living in poor urban areas. These programs should be cognizant of the fact that these individual level behavior factors are exacerbated by an environment affected by extreme poverty. Poverty has been considered a root cause of poor SRH outcomes especially in slum environments. Targeted multipronged interventions which address the economic, education and gender consideration of young people will therefore have the most direct influence on their behavior.

Although the Kenyan government has made significant strides in addressing the sexual and reproductive health (SRH) needs of young people, including passing significant policies such as the National School Health Policy, the implementation of this policy and its reach to young people is still low. The accelerated implementation of this and other youth related policies to include a comprehensive school health program to address youth SRH and broader health needs are of essence. Finally, finding innovative ways to demystify comprehensive sexuality education and looking for innovative delivery channels by which comprehensive sexuality education can be brought to schools is a necessity. Reaching young women in their early adolescence, while they are still in school, will have a potential of not only reducing risky sexual behaviors, but reduce unintended adolescent fertility, which will have significant impact on their wellbeing.
